# Advancing thermal performance through vortex generators morphing

**DOI:** 10.1038/s41598-022-25516-4

**Published:** 2023-01-07

**Authors:** Samer Ali, Talib Dbouk, Guanghui Wang, Dingbiao Wang, Dimitris Drikakis

**Affiliations:** 1 Univ. Lille, Institut Mines-Télécom, Univ. Artois, Junia, ULR 4515 – LGCgE, Laboratoire de Génie Civil et géo-Environnement, 59000 Lille, France; 2 CORIA, UMR 6614, CNRS, Normandy Univ., UNIROUEN, 76000 Rouen, France; 3 School of Mechanical and Power Engineering, Zhengzhou University, Zhengzhou, 450001 China; 4 Engineering Research Center of Energy Saving Technology and Equipment of Thermal Energy System, Ministry of Education, Zhengzhou, 450001 China; 5 University of Nicosia, 2417 Nicosia, Cyprus

**Keywords:** Fluid dynamics, Computational science

## Abstract

The design of rigid vortex generators (RVG) influences the thermal performance of various technologies. We employed Discrete Adjoint-Based Optimization to show the optimal development of vortex generators. Under turbulent flow conditions, different bi-objective functions on the RVG design were examined. Specifically, we aimed at an optimal RVG shape that minimizes the pressure drop and maximizes the local heat transfer in a rectangular channel. We show that an optimal design of an RVG can be obtained using computational fluid dynamics in conjunction with the Pareto Front at a computational cost of the order ~$O(10^{-1})$. We obtained three essential vortex generator shapes based on the RVG morphing technique. Compared to the baseline geometry of a delta winglet pair DWP, the first morphed design reduced the pressure drop by $39\%$, however, at the expense of a $21\%$ reduction in the Nusselt number. The second vortex generator design enhanced the heat transfer by $18\%$, however, at the cost of a significant increase in pressure drop of about $40\%$. The final morphed design achieved the highest thermal performance factor of 1.28, representing a heat transfer enhancement of $6\%$ with a moderate increase in pressure drop of about $13\%$ compared to DWP vortex generators. Furthermore, we investigated the effect of introducing different size holes on the mass reduction of vortex generators and their thermal performances. The mass of vortex generators can be reduced by $9\%$ and with an increase of $7\%$ in thermal performance factor concerning the DWP baseline. The findings of this study will lead to highly efficient lightweight heat exchangers.

## Introduction

Many industrial and engineering applications include conjugated heat transfer in complex fluid flow geometries such as multi-functional heat exchangers (HEX’s)^1^, reactors and mixers. However, the bi-objective optimization of conjugated heat transfer in channel flows of compact HEXs remains a computational challenge. Employing computational fluid dynamics (CFD) to identify a well-defined Pareto Front for optimal designs of fluid and thermo-fluid components is a time-demanding computational task because the design space is vast. The numerical studies usually compare variants of methods, e.g., adjoint versus adjoint-free parametric and shape optimization^2,3^ and topology optimization^4^. Thermo-fluid optimization in channels requires minimizing the overall pressure drop and maximizing the heat transfer.

Researchers developed different methods for enhancing heat transfer with vortex streets^5^, which can be classified as active, passive, and dynamic. Active techniques depend on external power sources, such as vibrations, oscillations or electromagnetic fields, aiming to perturb the flow locally. On the other hand, passive methods are more efficient and robust. They depend not on external sources, but local surfaces, such as deformation-induced perturbation, such as rigid vortex generators (RVGs)^3,6^. Finally, dynamic techniques for enhancing heat transfer employ elastically-deformed surfaces such as flexible vortex generators (FVGs)^7,8^.

The optimal design of RVGs is an active research topic^9^. During the last decade, progress in optimization methods and computational resources, e.g., cloud and high-performance computing, opened new perspectives of optimization in CFD. For example, adjoint-based optimization (ABO) methods^10^ employ adjacent variables to the state variable to estimate the objective function’s sensitivity field concerning the design variables. The sensitivity maps enter into a gradient-based optimization algorithm coupled with the CFD solver to find the overall objective function, e.g., employing a local deformation of the surface immersed in or surrounding the fluid flow.

Several researchers in the past performed computational modelling of vortical structures. For example, Danaila and Hélie^11^ investigated the post-formation evolution of a laminar vortex ring. Sun et al.^12^ studied the wake structure of a micro-ramp vortex generator in hypersonic flows. Tian et al.^13^ investigated different types of longitudinal RVGs in a flat plate channel flow to increase the heat transfer through the Nusselt number and reduce the pumping power through the friction factor. Moreover, researchers conducted numerical investigations of RVG designs like annular winglet; wave-element RVG^14,15^; curved winglet RVG with or without local perforations^16,17^; twisted and helical RVGs^18,19^; wavy-fins and oval-tube-bank with mounted RVGs^20,21^ and longitudinal vorticity RVGs^22^.

Zaman et al.^23^ tried experimentally controlling an axisymmetric jet by using vortex generators. Behfard and Sohankar^24^ characterized the local flow structures for a fluid flow in the presence of delta winglet pair (DWP) in a finned circular heat exchanger under a turbulent regime with a steady SST $k-\varepsilon$ turbulence model. Oneissi et al.^25,26^ investigated the influence of a new RVG design as an inclined-projected-winglet-pair (IPWP) on the heat transfer enhancement under a turbulent flow regime. Finally, Hamidouche et al.^27^ conducted flow characteristics downstream delta-winglet vortex generator experiments using the stereoscopic particle image velocimetry (PIV) technique in a rectangular channel but without heat transfer. Experimental measurements of RVGs with coupled fluid flow and heat transfer are scarce in the literature, and the last works go back to the 1990s by Fiebig and Tiggelbeck^28,29^. The difficulty is to cover a wide range of operating conditions, such as different Reynolds and Prandtl numbers, RVG designs and angles of attack.

The optimization of thermofluid components by employing advanced optimization techniques in CFD, e.g., heat transfer enhancement, pressure drop, and overall weight/mass reduction, has emerged as a topic of research and development. For example, Kim and Choi^30^ realized shape optimization of dimples RVGs in a rectangular channel by employing a Surface Response Optimization (SRO) method. Song et al.^31^ developed an advanced optimization technique in CFD using a Surrogate-Based Algorithm (SBA) for designing a heat sink. Recently, Karkaba et al.^3^ presented multi-objective function optimization through a large space exploration design developed to find optimal RVG design. They defined seven different design variables to draw/find the shape of the optimal RVG. A new enhanced RVG design was found recently by^3^ through parametric shape optimization. The optimal design^3^ led to a substantial increase in the thermal enhancement factor, though at the expense of computational costs. Moreover, Oh and Kim^32^ studied the thermal and flow characteristics of the rectangular-winglet (RW); delta-winglet-upstream (DWU); and delta winglet-downstream (DWD) curved vortex generators (CVGs). These designs varied regarding the position angle and the radial distance in the fin-tube heat exchanger configuration. Most of their results suggested that conventional VGs are superior in performance compared to curved vortex generator CVGs. Carpio and Valencia^33^ investigated the effect of introducing longitudinal vortex generators (LVGs) in arrays consisting of 9 alternating, 18 alternating, 18 aligned, 27 alternating and 39 alternating LVGs. Results show that the design with the best thermal performance corresponds to geometry with 39 LVG, with a $52\%$ increase compared to the flat fin geometry. The case with an enhanced performance corresponds to the geometry with 18 vortex generators, which presents a performance increase of $38\%$ to the baseline design. Therefore, it performs similarly to the 39 LVG case, despite having only half the vortex generators. Khan et al.^34^ adopted artificial neural networks (ANNs) for predicting the efficiency of a double-pipe heat exchanger which employs T-W tape inserts with different wing-width ratios. The ANNs predicted with high accuracy thermal parameters such as the friction factor, the Nusselt number and the thermal performance factor (TPF) of a double pipe heat exchanger. More recently, Khan et al.^35^ used ANN to optimise the performance of a double pipe heat exchanger, i.e., to obtain better heat transfer enhancement with a low-pressure drop penalty. They found a maximum value of TPF equal to 1.44 by varying five input parameters related to wing-width ratio, pitch ratio, attack angle, Reynolds number, and tube length (L).

As seen in the research mentioned above, the design method for vortex generators typically revolves around a set of geometrical parameters such as the angle of attack, inclination angle, span, height, and different shapes such as delta wing and rectangular wing. Since the parametric space is ample, it becomes a tedious task to define a concrete parametric optimization problem without considering that designing vortex generators within these parameters is somewhat restrictive. Optimization processes typically require extensive CFD calculations to explore the variations of just a few parameters. Therefore, effective optimization techniques are needed to rapidly advance the development of vortex generators’ performance. To this end, gradient-based optimization methods aim to find ’sensitivity gradients’ from the solution of the flow field. These will indicate how the performance will change by modifying the vortex generator’s geometry. Such advanced techniques, therefore, minimize the computational cost of the optimization process. Gradient-based methods have been developed mainly for aerospace applications to enhance lift and reduce drag for airfoils geometries^36,37^ but also have great potential for heat exchangers technology. The present work chooses the Adjoint method for several reasons. To the author’s knowledge, Adjoints have not been used in conjunction with vortex generators in current literature. Still, they can be powerful and efficient tools in developing vortex generator-based heat exchangers. Furthermore, popular CFD codes such as ANSYS Fluent have an Adjoint solver already implemented so that the methods developed here are widely accessible to the general CFD/Thermal engineering community working on vortex generators. This will increase the impact of this research and shed more light on the Adjoint method’s applicability to heat enhancement techniques based on the vortex generator technology.

In this study, we employ an adjoint-based shape optimization method in conjunction with CFD for the optimal and rapid design of RVGs in a turbulent flow regime. We define a bi-objective Adjoint-Based Optimization problem to compute the sensitivity maps by: (1) locally morphing the mesh and deforming the RVG surface topology in a controlled volume sub-domain; (2) creating the overall optimization algorithm to act in an enhanced and more rapid optimal line-search direction using the steepest descent algorithm, thus reducing the overall computational time of the Pareto Front by an order ~$O(10^{-1})$. For the first time, we show and quantify the direct influence of the mathematical formulation of the BOF on the Pareto Front, the overall computational time and the RVGs design optimality.

## Results

**Figure 1:**
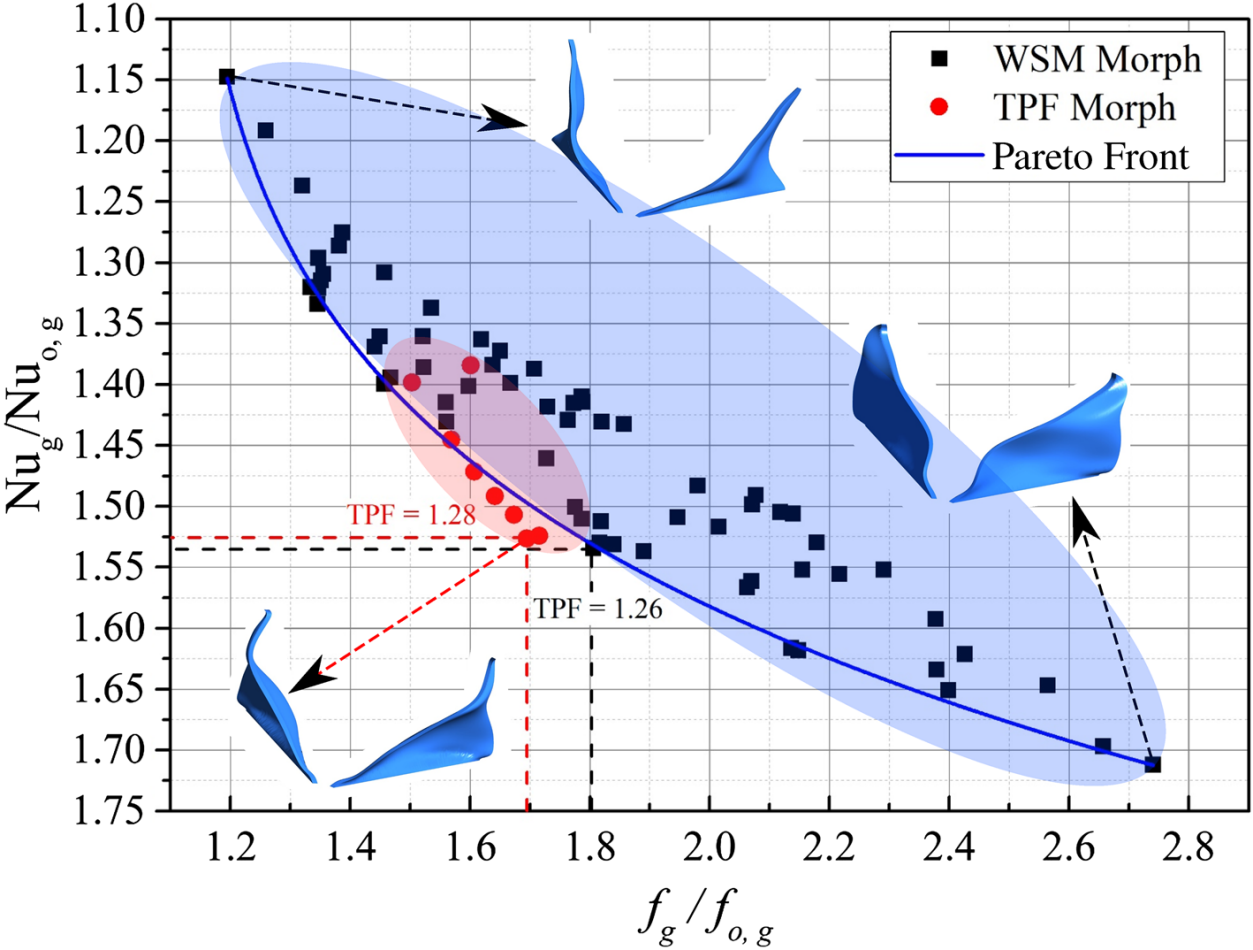
Pareto Front of the optimum designs of RVG using adjoint-based optimization employing two different bi-objective functions: $f_1$ (TPF Morph based, Eqs. 4, 9); $f_2$ (WSM Morph based, Eq. 5). The overall computational time to obtain the TPF morph-based optimum design is reduced tenfold compared to the WSM. This reduction in computational time can be explained by the reduction of the design space of the exploration represented schematically by the two coloured zones in red (for $f_1$) and blue (for $f_2$). The optimal RVG design obtained by $f_1$ has $TPF=1.28$ and the optimal RVG design obtained by $f_2$ has $TPF=1.26$.

The Pareto Front for the RVG designs fits well with a Pareto function (Fig. 1). The solid squares in Fig. 1 represent the adjoint-based designs obtained with a weighted sum of function (WSM) $f_2$ (Eq. 5) while the solid circles represent the adjoint-based RVG designs obtained with thermal performance factor (TPF) morph function $f_1$ (Eqs. 4, 9). The design space using $f_2$ is much larger than the design space using $f_1$ represented by the coloured elliptical schematic zones in Fig. 1. Therefore, using the ABO algorithm with BOF in the form of $f_1$ (TPF morph) can find optimal designs more efficiently and rapidly than BOF in the form of $f_2$ (WSM Morph). The overall computational time to obtain the TPF morph-based optimum ($TPF=1.28$) design is reduced tenfold compared to the WSM morph-based optimum design ($TPF=1.26$). Increasing the *TPF* above one, even by a small percentage of $1\%$, gives a major improvement when optimizing a single RVG in a channel flow. A minimal increase in the *TPF* value, e.g., $1\%$, can have a much larger enhancement in a compact heat exchanger (HEX) overall performance due to thousands of locally distributed RVGs on the HEX channel surfaces.

**Figure 2:**
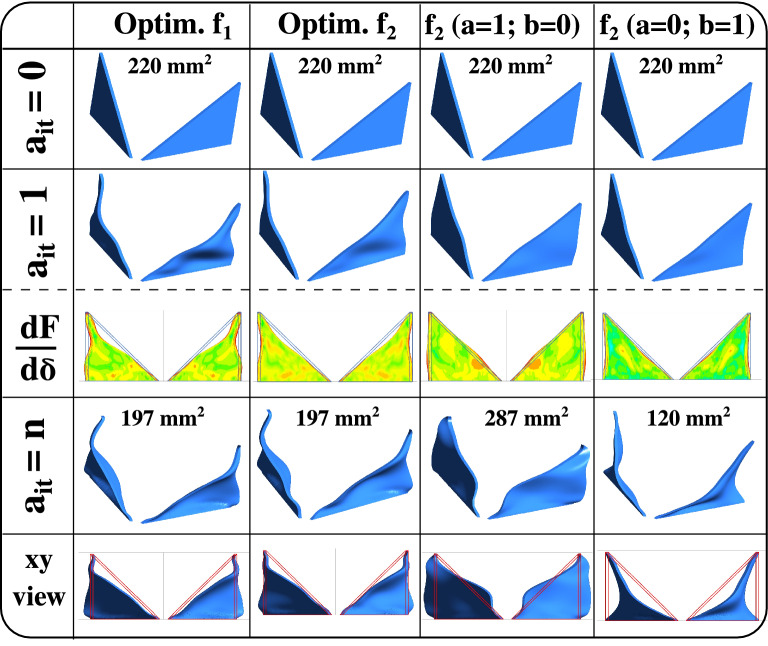
Optimization results showing designs evolution as function of the adjoint iterations $a_{it}$ in the ABO algorithm (Fig. 10). The RVG shape change showing an example of the sensitivity $\frac{dF}{d \delta }$ maps after the first adjoint iteration $a_{it}=1$. Comparisons between the DWP initial base design at $a_{it}=0$ and the three adjoint-based optimum designs obtained with a TPF Morph objective $f_1$, extreme objective $f_2~(a=1;b=0)$ and extreme objective $f_2~(a=0;b=1)$. The frontal area of RVG in the xy-plane is shown in $\mathrm{{mm}}^2$ at the beginning at $a_{it}=0$ and the end at $a_{it}=n$ of the ABO iterations (see the algorithm in Fig. 10).

The RVG’s shape evolution during the ABO process (algorithm of Fig. 10) is shown in Fig. 2. We also show the frontal area evolution in $\mathrm{{mm}}^2$ and the different RVG designs initially at $a_{it}=0$, after the first adjoint iteration $a_{it}=1$ and at the end of the overall iterations $a_{it}=n$. From Fig. 2, we observe that the ABO algorithm, using TPF morph BOF $f_1$, succeeds in deforming the RVG surface in a way that promotes the local creation or generation of additional vortices. This can be seen from the sharp edge at the upper lateral corners of the optimal RVG. Moreover, the frontal area is reduced from 220 to $197~\mathrm{{mm}}^2$ in optimal designs obtained by $f_1$ and $f_2$ compared to the initial DWP design.

**Figure 3:**
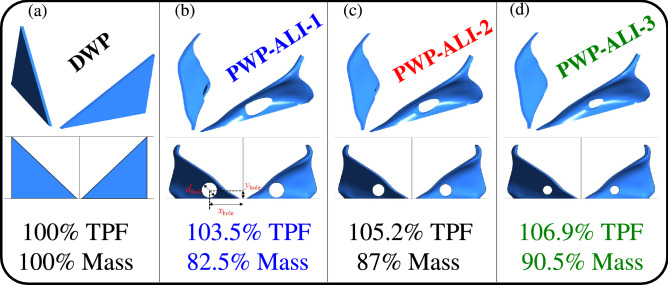
Optimization results for the TPF values versus mass reduction of the three optimal perforated PWP RVG designs, compared with the reference DWP RVG design that has $100\% Mass$ and $100\% TPF$ with $TPF=1.16$. According to the DWP reference design, an empty channel will have only $84\% TPF$ with $TPF=1$. PWP were perforated with different hole diameters $d_{hole}$ such that: (**b**) $d_{hole}=5~\mathrm{{mm}}$, (**c**) $d_{hole}=4~\mathrm{{mm}}$, (**d**) $d_{hole}=3~\mathrm{{mm}}$. The position of the hole is: $x_{hole}=11~\mathrm{{mm}}$ and $y_{hole}=2.8~\mathrm{{mm}}$.

Here we give specific attention to optimizing the mass of the optimal RVG design. As shown in Fig. 3, we investigate the mass reduction of the new RVG design by introducing different holes at a time, each of diameter $d_{hole}$. Compared to DWP RVG, it is found that we can reduce the mass as in the new perforated RVG by $9.5\%$ with a TPF increase of about $7\%$, only at the scale of a single RVG (see PWP-ALI-3 in Fig. 3). Nevertheless, more significant mass reduction is associated with a lower increase in the TPF, which is seen in Fig. 3, where the mass can be reduced by up to $17.5\%$, however, with a smaller increase of $3.5\%$ in TPF when compared to the DWP RVG. So, more HEX designs can be produced at a larger scale using the new PWP-ALI RVG design within a turbulent flow in rectangular channels. As a result, the future HEX can have highly reduced overall mass and a good increase in its overall TPF. This is a significant finding in research and development of turbulent fluid flow in rectangular channels and its effect on designing optimal compact heat exchangers/reactors of reduced mass versus increased overall TPF. By taking a plate-fin heat exchanger with inserted vortex generators made of Aluminum material that has a density of $2700~{\mathrm{{kg}}/\mathrm{{m}}^3}$, the mass of one pair of DWP RVGs will be equivalent to approximately 2.16 g. Based on the results of Tiggelbeck et al.^38^ where two rows of DWP RVGs can be inserted along a channel length of 15*H* for effective heat transfer enhancement. As a result, this will correspond to a mass per volume ratio of 7200 ${\mathrm{{g}}/\mathrm{{m}}^3}$, i.e. 7200 g of DWP RVGs mass per one cubic meter of heat exchanger volume. The results for the amount of mass reduced per unit cubic meter of heat exchanger volume for the three perforated designs PWP-ALI-1,2 and 3 are summarised in Table 1.

**Table 1:** Amount of mass reduced for PWP RVGs compared to DWP RVG.

RVG design	Mass of RVG in ${{\mathrm {g}}/{\mathrm {m}}^3}$	Reduced mass in ${{\mathrm {g}}/{\mathrm {m}}^3}$
DWP	7200	–
PWP-ALI-3	6516	684
PWP-ALI-2	6264	936
PWP-ALI-1	5940	1260

## Discussion

Figure 3b–d illustrate the local surface deformation induced by the adjoint-based optimization algorithm to maximize the TPF and minimize the overall mass. One can observe that the initial geometry (DWP) is locally deformed at different height positions from $y=1~\mathrm{{mm}}$ to $y=19~\mathrm{{mm}}$ concerning the bottom wall at $y=0$ where the RVG is fixed. Between $y=1~\mathrm{{mm}}$ and $y=10~\mathrm{{mm}}$, which corresponds to half the channel’s height, the ABO algorithm deforms the surface of the DW RVG towards the lateral side away from the centre-line of the main flow. Above $y=12~\mathrm{{mm}}$ and close to the upper wall, the ABO algorithm deforms the initial RVG design towards the centre-line of the main flow. This reversed curvature of the RVG surface has an essential feature in fluid flow physics. It permits the production of additional local vortices, thus enhancing heat recovery from the lateral walls at a reduced pressure drop in the channel.

**Figure 4:**
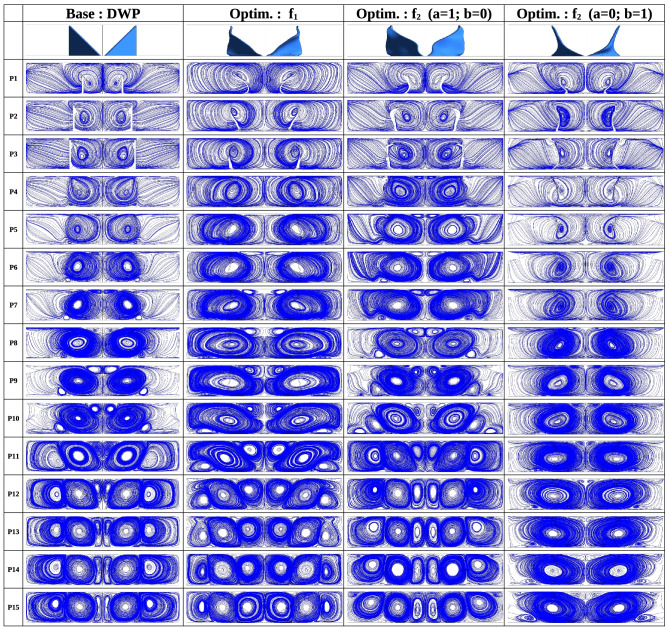
Streamwise velocity Streamlines at different *z*/*L* positions downstream the RVG for: Base DWP design, Optimum design using $f_1$ (Eqs. 4, 9), Optimum design using $f_2$ with $a=1$ and $b=0$ (Eq. 5) and Optimum design using $f_2$ with $a=0$ and $b=1$ (Eq. 5).

**Figure 5:**
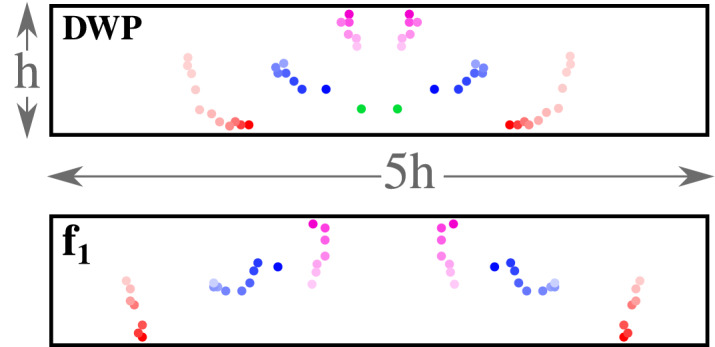
Local dynamics of the vortical centers from inlet to outlet for the initial DWP design and the optimized design obtained using a TPF Morph objective function $f_1$ (see Eqs. 4, 9). The colour intensity of the vortical centres decreases progressively as going in the stream directly from the inlet to the outlet.

Flow structure and temperature distribution are also analysed to understand better the heat transfer enhancement mechanism by local RVGs. Figure 4 shows the velocity streamlines along 15 positions from P1 to P15. The streamlines are computed in 15 different xy-planes distributed with equal spacing between the inlet ($z=0$, P1) and outlet ($z=15h$, P15). Between P1 and P7 in all the RVG designs, two major vortices are created downstream of the RVG. Looking at the plane P7, the two vortices generated by the BOF $f_1$ are clearly wider than those produced by the DWP RVG and the two extreme conditions with $f_2$ ($a=1;~b=0$) and $f_2$ ($a=0;~b=1$). Between P9 and P15, the optimal RVG design obtained with $f_1$ develops six local longitudinal vortices. The latter have vertical centres closer to the channel’s lateral boundaries; see the red-coloured solid circles in Fig. 5, which represent the local dynamics of the vortical centres from inlet to outlet. Compared to the DWP design, the optimal RVG obtained with $f_1$ produces three major vortical structures more displaced downstream in the XY lateral directions. This removes more heat from the walls by forced convection and at a reduced pressure drop.

**Figure 6:**
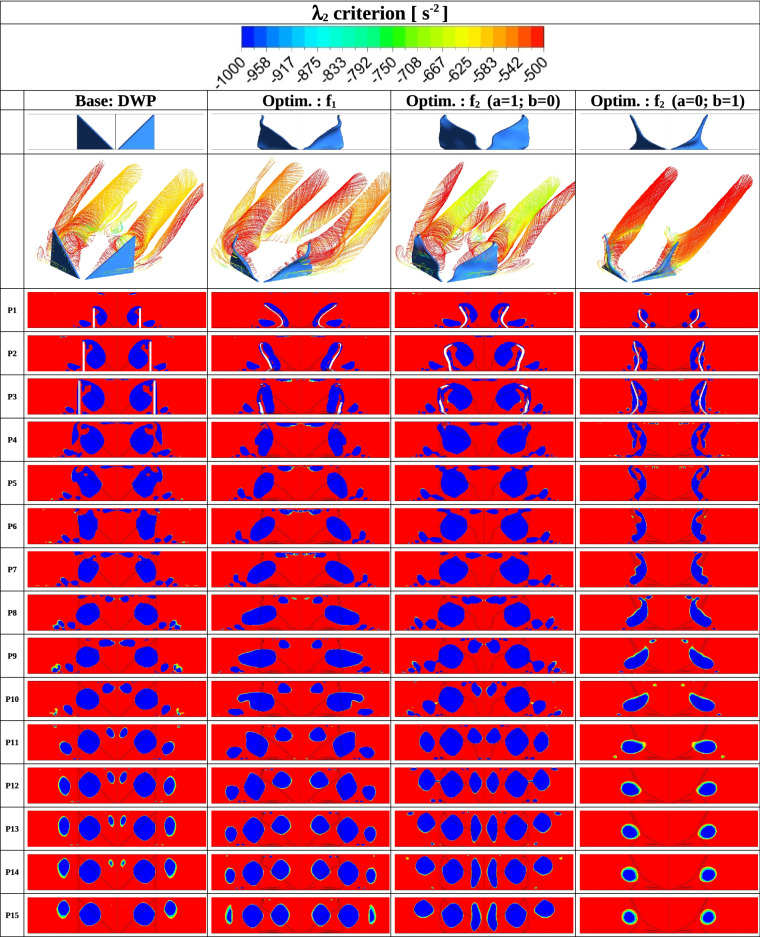
$\lambda _2$ criterion fields for: Base DWP design, Optimum design using $f_1$ (Eqs. 4, 9), Optimum design using $f_2$ with $a=1$ and $b=0$ (Eq. 5) and Optimum design using $f_2$ with $a=0$ and $b=1$ (Eq. 5).

To better investigate the turbulence production rate in the channel due to the RVGs, we compute in Fig. 6 the $\lambda _2$-criterion. At P15 with optimal $f_1$, it can be observed that six major vortices have better conserved strength and more uniform spanwise distribution compared to DWP and the two extreme conditions when using the BOF $f_2$ with $a=1$  and  $b=0$ and with $a=0$  and  $b=1$.

**Figure 7:**
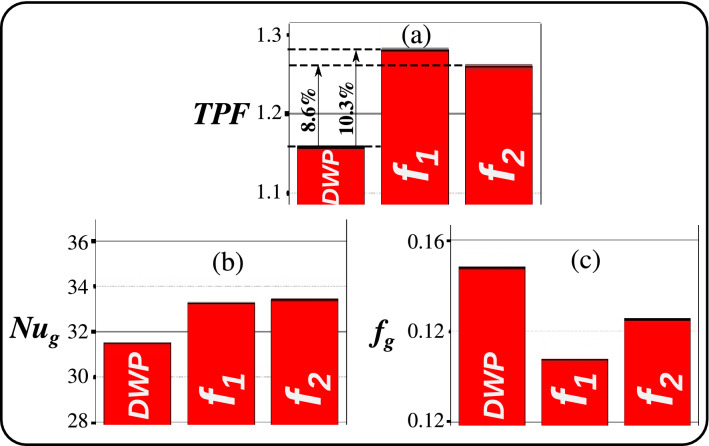
Optimization results of: (**a**) thermal performance factor *TPF* (Eq. 4). (**b**) Global Nusselt number $\mathrm{{Nu}}_g$. (**c**) Global friction factor $f_g$. The DWP base design and the two adjoint-based optimum designs were compared with a TPF morph-based objective $f_1$ and a WSM-based objective $f_2$. The TPF Morph optimal design is obtained at a computational cost reduced by a factor of 10, compared to the WSM optimal design.

In terms of quantitative analysis of fluid flow physics and heat transfer, we compute the *TPF* (see Eqs. 4, 9), global Nusselt number $\mathrm{{Nu}}_g$ and global friction factor $f_g$. Figure 7 clearly shows that the *TPF* value is increased by about $8.6\%$ when using a WSM morph-based BOF $f_2$, while the *TPF* value is increased by $10.3\%$ when employing a TPF Morph based BOF $f_1$. Moreover, the TPF Morph optimal design is obtained at a computational cost reduced by a factor of 10, compared to the WSM optimal design. This illuminates the advantages of employing ABO with BOF of the form of $f_1$ (Eqs. 4, 9) rather than of the form of $f_2$ (Eq. 5).

For further local analysis of the flow in the channel, the readers may refer to the supplementary information document attached to the manuscript.

In conclusion, we employed the Discrete ABO method coupled with advanced CFD to optimise a DWP RVG under a turbulent flow regime at $\mathrm{{Re}} \approx 4600$. The objective is to increase the heat transfer and reduce the pressure drop in a rectangular channel. In other words, we seek to improve the TPF considerably. For that, we formulated two forms of a BOF with attention to the overall mass of the RVG: (1) a TPF Morph function; (2) a weighted summation method or function (WSM) with two weighting factors. We quantitatively showed how one could obtain an optimal design of a local RVG in a channel on a Pareto Front with a very well-reduced computational time of order ~$O(10^{-1})$. The above is achieved using a TPF morph BOF within an ABO method in CFD. We showed how the ABO algorithm, coupled with the CFD solver, conducted a local surface morphing of the initial RVG design with more local surface deformation at the corner side of the VG. This ensured that the new RVG produced more local vortices towards the lateral sides of the channel. As a result, the ABO algorithm converged toward a new design of RVG, which increased the thermal performance factor (TPF) by $10.3\%$. Furthermore, we investigated the mass reduction of this new RVG by adding a hole. Different hole diameters were investigated. Compared to a DWP RVG, we found that the mass of the new Perforated Winglet Pair RVG (named “*PWP-ALI*”) is reduced by $9.5\%$, with TPF increased by about $7\%$. The above implies that, at larger scales, new HEX designs can be produced using PWP-ALI RVG so that the overall HEX can have a primarily reduced mass (presence of thousands of RVGs). An essential increase in thermal performance will accompany this compared to the DWP RVG design. Finally, this work showed the importance of ABO methods applied in CFD compared to other methods. Furthermore, the numerical methodology and the present findings open new perspectives for ABO techniques in CFD for finding optimal designs in a more robust, fast and reliable manner, e.g. compared to parametric and shape optimization methods in CFD.

## Methods

### Geometry and computational domain

**Figure 8:**
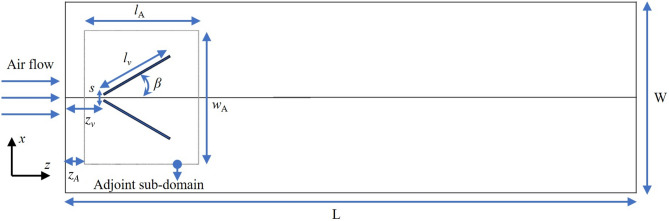
The geometry of the rectangular channel with delta-delta winglet pair. The adjoint shape optimization problem is solved in the adjoint sub-domain. Dimensions of the channel are $h=20~\mathrm{{mm}}$, $W=5h$ and $L=15h$. The RVG has $s=0.2h$, $z_v=h$ and $\beta =30^{\circ }$. The adjoint sub-domain has a control volume $\Omega _v=I_A \times w_A \times h$ with the following dimensions: $I_A=7h/2$, $w_A=7h/4$. It is positioned at $z_A=h/2$ away from the inlet enveloping the RVG to be morphed.

The computational three-dimensional (3D) domain is made of a channel of a rectangular form (see Fig. 8) of height $h = 20~\mathrm{{mm}}$, width $W=5h$, and length $L=15h$ similar to the experimental DWP case by Tiggelbeck et al.^29^. The distance from the inlet to the RVG tip is denoted by $z_v$ with $z_v=h$. The angle of attack is $\beta =30^{\circ }$ and the spacing between the RVG tips is $s=0.2h$. The span length of the RVG is $l_v=2h$. The adjoint sub-domain in Fig. 8 is at same channel’s height *h* and has the following dimensions: $I_A=7h/2$, $w_A=7h/4$ with a control volume $\Omega _v=I_A \times w_A \times h$. It is placed at $z_A=h/2$ away from the inlet, thus completely enveloping the RVG surface to be morphed by the ABO algorithm.

### Governing equations and boundary conditions

The fluid flow is incompressible and turbulent inside the rectangular channel at a Reynolds number of $\mathrm{{Re}} \approx 4600$. This specific value of the Reynolds number is chosen because it corresponds to a maximum TPF value for the RVG compared to other higher or lower values of Re, as was shown in the literature by Oneissi et al.^25^. The conjugated fluid dynamics and heat transfer are solved through the following consecutive mass, momentum and energy conservation equations:

1 $$\begin{aligned}&\nabla \cdot \mathrm {{\textbf {U}}} = 0 \end{aligned}$$

2 $$\begin{aligned}&\rho \frac{\partial \mathrm {{\textbf {U}}}}{\partial t} + \rho \nabla \cdot [ \mathrm {{\textbf {U}}} \mathrm {{\textbf {U}}} ] = \nabla \cdot \Big [ (\mu +\mu _t) \nabla \mathrm {{\textbf {U}}} \Big ] \end{aligned}$$

3 $$\begin{aligned}&\rho C_{p} \frac{\partial T}{\partial t}+ \rho C_{p} \nabla \cdot {[}\mathrm {{\textbf {U}}} T ] = C_{p} \nabla \cdot \Big [ \Big (\frac{\mu }{\mathrm{{Pr}}}+\frac{\mu }{\mathrm{{Pr}}_t} \Big ) \nabla T \Big ] \end{aligned}$$

where ${\textbf {U}}$ is the velocity vector, *T* the temperature, $\rho$ the fluid density, $\mu$ the fluid’s dynamic viscosity, $\mu _t$ the turbulent dynamic viscosity, *Cp* the specific heat, Pr and $\mathrm{{Pr}}_t$ are the Prandtl and turbulent Prandtl numbers, respectively. The operating fluid is air.

The Reynolds averaged Navier–Stokes (RANS) in conjunction with the $k-\omega -SST$ (Shear-Stress transport) model^39^ are employed to solve for the turbulent flow field. The SST model includes two additional partial differential equations coupled to 1, 2 and 3 to account for the turbulent kinetic energy *k* and the turbulent dissipation rate $\omega$. The SST model employs a $k-\omega$ model formulation in the inner parts of the boundary layer and the $k-\varepsilon$ model in the freestream. The $k-\omega -SST$ model has shown accurate predictions and numerical convergence for flows around RVGs^26^.

**Figure 9:**
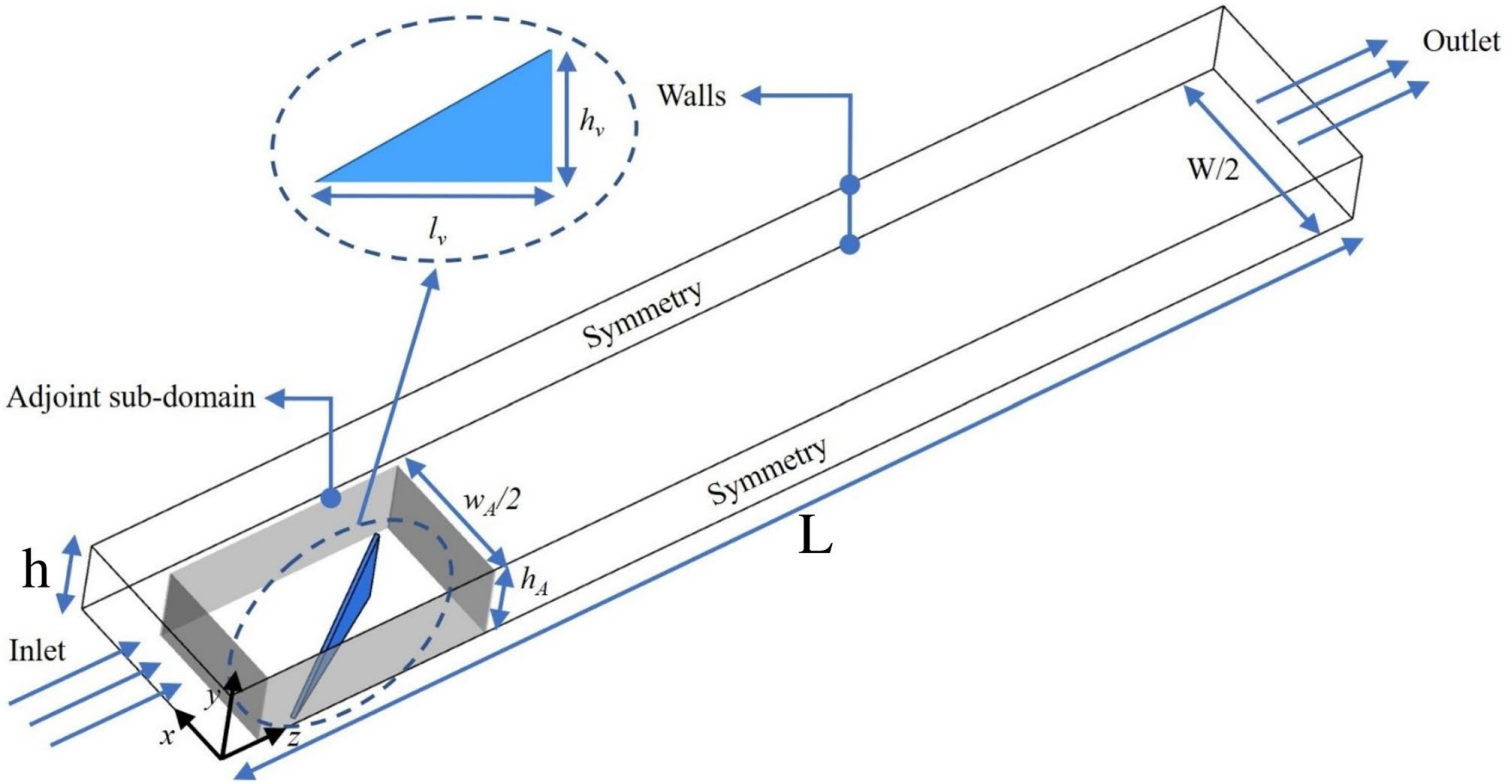
Computational domain showing the boundaries of the channel. Velocity inlet and pressure outlet are imposed respectively at the inlet and outlet. Symmetry is imposed at the external boundaries. Rigid walls with no-slip velocity boundary conditions are imposed on the RVGs surface and on top and on bottom of the channel. Constant uniform temperature $T_w$ is imposed on top and on bottom walls while an adiabatic boundary condition is applied at the RVG surface.

The boundary conditions (Fig. 9) are set as follows

- Inlet: A constant temperature $T_{in}$ and uniform velocity at the inlet $U_{in}$.
- Upper and lower channel surfaces: a constant temperature $T_{w}$.
- Left and right channel surfaces: a symmetry boundary condition.
- VG surface: adiabatic wall with zero heat flux.
- Outlet: a zero-pressure Neumann boundary condition.

### Adjoint-based optimization in CFD

**Figure 10:**
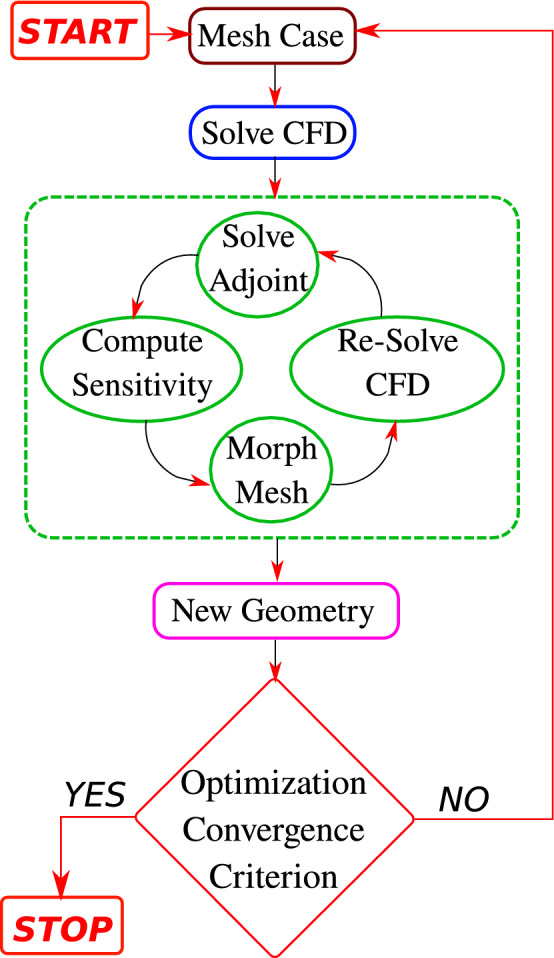
The Computational Algorithm of the Adjoint and CFD Solvers with Mesh Morphing technique. The optimization convergence criterion is based on a steepest descent gradient-based optimization sub-algorithm.

The flow chart of the adjoint-based optimization (ABO) algorithm is shown in Fig. 10, and the components of ABO are discussed below.

#### Discrete adjoint method

Adjoint optimization methods require no parameterization of the geometry, which is the case in other methods, such as in parametric optimization techniques in CFD^3^. Moreover, adjoint-based methods can produce better engineering designs at an overall reduced cost. Compared to the continuous adjoint method, the discrete approach computes the sensitivity field more accurately, especially in turbulent flow conditions.

#### Optimization problem

Two mathematical problems of adjoint-based optimization are formulated and solved by minimizing two different global bi-objective functions (BOF) $f_1$ and $f_2$ as the following:

4 $$\begin{aligned} f_1&= -TPF \end{aligned}$$

5 $$\begin{aligned} f_2&= -a \left( {\frac{\mathrm{{Nu}}_g}{\mathrm{{Nu}}_{0,g}}}\right) + b \left( {\frac{f_g}{f_{0,g}}}\right) \end{aligned}$$

where $f_g$ is the global friction factor, $\mathrm{{Nu}}_g$ the global Nusselt number, and the subscript “0” denotes an empty channel. $f_1$ is a linearly weighted sum morph (WSM) objective function with two real parameters *a* and *b* such that $a,b \in [0-1]$. $f_2$ is a ratio form single objective defined as a TPF Morph based function.

The global Nusselt number is calculated using the logarithmic mean temperature difference method as follows:

6 $$\begin{aligned} \mathrm{{Nu}}_g=\frac{q^{''}_w}{LMTD} \frac{D_h}{k} \end{aligned}$$

where $q^{''}_w$ is the average wall heat flux at the channel top and bottom walls, $D_h$ is the hydraulic diameter of the channel and is equal to 2*h* and *k* is the thermal conductivity of the flowing fluid.

The log mean temperature difference *LMTD* is calculated using the following equation:

7 $$\begin{aligned} LMTD=\frac{\Delta T_i-\Delta T_o}{\ln \bigg (\Delta T_i/\Delta T_o\bigg )} \end{aligned}$$

where $\Delta T_i$ is the temperature difference between the wall and the average temperature at the channel’s inlet and $\Delta T_o$ is the temperature difference between the wall and the average temperature at the channel’s outlet, respectively.

Moreover, the global friction factor $f_g$ is related to the pressure drop $\Delta p$ between the inlet and the outlet of the channel by:

8 $$\begin{aligned} f_g=\frac{\Delta p}{\frac{1}{2} \rho U^2_{in}} \frac{D_h}{L} \end{aligned}$$

where $U_{in}$ is the air velocity at the inlet of the channel. The thermal peformance factor (TPF) of Eq. (4) is given by:

9 $$\begin{aligned} TPF = \bigg (\frac{\mathrm{{Nu}}_g}{\mathrm{{Nu}}_{0,g}}\bigg ) \bigg (\frac{f_g}{f_{0,g}}\bigg )^{-1/3} \end{aligned}$$

To analyze the optimized RVGs performance, the local Nusselt number inside the channel is computed as a function of the dimensionless local position *z*/*h* in the flow direction:

10 $$\begin{aligned} \mathrm{{Nu}}_{z}=\frac{h_{z} D_{h}}{k} \end{aligned}$$

where

11 $$\begin{aligned} h_{z}=\frac{{q^{''}_{z}}}{T_{w}-T_{z}} \end{aligned}$$

and

12 $$\begin{aligned} {T_{z}} = \frac{\int U \ T \ dA}{\int U \ dA} \end{aligned}$$

where $T_{z}$ is the local temperature, $T_{w}$ is the wall temperature, $D_h$ the hydraulic diameter, ${q^{''}_{z}}$ is the local wall heat flux, $Nu_{z}$ the local Nusselt number and $h_{z}$ the local heat transfer coefficient.

The local friction factor (or fanning friction factor) in the channel $f_{z}$ is computed as the following:

13 $$\begin{aligned} f_{z} = \frac{\tau _w}{{\frac{1}{2} \rho \ U^{2}_{in}}} \end{aligned}$$

where $\tau _w$ is the shear stress at the wall.

Finally, the Reynolds number is calculated based on the hydraulic diameter of the channel as follows:

14 $$\begin{aligned} \mathrm{{Re}}=\frac{\rho U_{in} D_h}{\mu } \end{aligned}$$

#### Sensitivity maps and mesh morphing control

For computing the shape sensitivity of the RVG concerning the objective function *F* such that $F \in [f_1,~f_2]$, we denote $\delta$ as the Cartesian coordinates (X, Y, Z) locations for every cell or node in our computational domain in the control volume $\Omega _v=I_A \times w_A \times h$ (see Fig. 8) enveloping the RVG surface. Thus, $\delta$ is an input vector to the overall adjoint optimization problem such that:

15 $$\begin{aligned} \Big [ \frac{dF}{d \delta } \Big ] = \Big [ \frac{\partial F}{\partial \delta } \Big ] + {\lambda _m^T} \Big [ \frac{\partial R}{\partial \delta } \Big ] \end{aligned}$$

The first term $\frac{dF}{d \delta }$ represents the total sensitivity of *F* with respect to $\delta$ at a given cell center or node. This is required to perform the mesh morphing and apply an optimal iterative displacement to the RVG surface (in outward or inward direction, depending on the sign) to satisfy the minimization of *F*. A mesh morphing algorithm guarantees the quality of the morphed mesh through local control of the positive volume of each finite volume cell. The steepest descent algorithm guarantees the convergence of the optimization problem toward a local minimum of *F* in the design space. The second term $\frac{\partial F}{\partial \delta }$ corresponds to the change in *F* due to a change in the position at a given cell center. The final term $\frac{\partial R}{\partial \delta }$ is the change in *F* due to the sensitivity of the flow solution to changes in the local position of the RVG. *R* is also known as the Residuals vector/matrix of the flow solution, and $\lambda _m^T$ is a vector of Lagrange multipliers, representing the adjoint solution variables, that is found by satisfying:

16 $$\begin{aligned} \Big [ \frac{\partial R}{\partial \beta }\Big ]^T \lambda _m = - \Big [ \frac{\partial F}{\partial \beta }\Big ]^T \end{aligned}$$

Equation 16 is also known as the adjoint problem that is solved using the same fluid flow solvers, and $\beta$ is the vector of fluid flow solution variables.

Polyhedral cells are generated in the computational domain employing a local mesh refinement method and a volume control imposed in the VG design space zone. The automatic local refinement algorithm ensures that the value of the dimensionless wall distance $y^+$ in the wall-bounded channel flow always satisfies $y^+ \le 1$ during the iterative procedure of mesh morphing.

Several polyhedral grids have been generated to choose the appropriate mesh size in a mesh sensitivity analysis procedure. The grid-convergence index (GCI) chooses the appropriate initial mesh size. A polyhedral mesh which contains $1.3 \times 10^{6}$ cells is adopted with less than $2 \%$ GCI value^40^ validated with the experimental DWP design of Tiggelbeck et al.^29^ at same Reynolds number $\mathrm{{Re}}=4600$. The comparison between numerical simulations and experimental results is summarized in Table 2 based on the calculation of the Nusselt number normalized by the Nusselt number corresponding to an empty channel and a normalized friction factor and the thermal performance factor (TPF). The results in the table show that the numerical simulation model agrees with the experimental results done by Tiggelbeck et al.^29^ with an error that is less than $3.5\%$.

**Table 2:** Normalized global Nusselt number, friction factor and thermal performance factor comparison between present numerical simulations and experimental results at Re = 4600.

	Experiment	Present CFD results	Error ($\%$)
$\mathrm{{Nu}}_g/\mathrm{{Nu}}_{o,g}$	1.49	1.45	2.7
$f/f_{o,g}$	1.91	1.95	2.2
TPF	1.2	1.16	3.4

Another approach to verify the choice of a numerical model and mesh size is the validation with fully developed turbulent flow in an empty channel. The numerical results are compared with the correlation of Dittus–Boelter^41^ for the evaluation of the global Nusselt number and the correlation of Blasius^42^ for the assessment of the global friction factor. The CFD results for an empty channel compared to the empirical correlations show an error of less than 2% for both the global Nusselt number and the friction factor.

In each ABO inner loop, the convergence of the CFD solvers is verified by looking at the friction factor *f* and Nusselt number (Nu) stability versus sub-iterations.

A mesh strategy in each ABO outer loop is set as follows: a control volume $\Omega _v$ is defined as an internal subdomain (Fig. 8) for the local deformation (mesh morphing) of the RVG surface based on the iteratively computed sensitivity map/field $\frac{dF}{d \delta }$ (see Fig. 10).

## Supplementary Information

Supplementary Information.

## Data Availability

Data is available upon request from the corresponding authors.
