# Supplementary material for: Advancing thermal performance through vortex generators morphing
Source: Sci Rep. 2023 Jan 7;13:368. doi: 10.1038/s41598-022-25516-4 (PMC9825620; doi:10.1038/s41598-022-25516-4)
Supplement: Supplementary file 1 — Supplementary Information. [file 41598_2022_25516_MOESM1_ESM.pdf]

Advancing Thermal Performance through Vortex Generators Morphing

Samer Ali[1,*], Talib Dbouk[2,*], Guanghui Wang[3,4], Dingbiao Wang[3,4], and Dimitris Drikakis[5]

[1]Univ. Lille, Institut Mines-Télécom, Univ. Artois, Junia, ULR 4515 – LGCgE, Laboratoire de Génie Civil et géo-Environnement, F-59000 Lille, France
[2]CORIA, UMR 6614, CNRS, Normandy Univ., UNIROUEN, 76000 Rouen, France
[3]School of Mechanical and Power Engineering, Zhengzhou University, Zhengzhou 450001, China
[4]Engineering Research Center of Energy Saving Technology and Equipment of Thermal Energy System, Ministry of Education, Zhengzhou 450001, China
[5]University of Nicosia, Nicosia, CY-2417, Cyprus
[*]Corresponding authors emails: email@talibdbouk.com; samer.ali@junia.com

Supplementary Information

Mathematical Model of the Adjoint-based Constrained Optimization Problem

Let us denote δ, the vector of all parameters that define the vortex generator (VG) design, e.g., the locations of the grid nodes that define the VG's shape. Thus, the cost function $\mathcal{F}$ depends on these design parameters but also on the flow conditions (state variables) $\mathcal{G}(\delta)$[1,2] such that:

$$\mathcal{G}(\delta) = \mathcal{G}(\mathbf{U}, p, T) \tag{1}$$

$$\mathcal{F} = \mathcal{F}(\mathcal{G}(\delta), \delta) \tag{2}$$

$\mathbf{U}$ denotes the velocity vector $(\mathbf{U}) = (U_x, U_y, U_z)$, p the pressure and T the temperature.
For solving the equations of the flow and design optimization, we should satisfy the equality constraint given by:

$$\mathcal{R}(\mathcal{G}(\delta), \delta) \ = \ 0 \tag{3}$$

As the state variables experience a slight change ∂ and the design (control) variables, δ, alter, the objective function, $\mathcal{F}$, also changes as follows:[3]

$$\partial \mathcal{F} = \frac{\partial \mathcal{F}}{\partial \mathcal{G}} \partial \mathcal{G} + \frac{\partial \mathcal{F}}{\partial \delta} \partial \delta \tag{4}$$

Note that changes of $\mathcal{F}$ are responses to changes in $\mathcal{G}$ and δ, thus a change in the geometry or other boundary conditions (terms of equation 4). The Adjoint-based method allows the approximation of the first term of the equation 4 at a low computational cost. The mathematical expression depends only on δ. Moreover, any change in the flow field imposes $\partial \mathcal{R}$ being zero.

Lagrange multipliers $\boldsymbol{\lambda}$ are introduced to decouple the constraints from the objective function. This is needed to transform the constrained optimization problem into an unconstrained one:

$$\mathcal{L} \ = \ \mathcal{F}(\mathcal{G}, \delta) \ + \ \boldsymbol{\lambda}^T \mathcal{R}(\mathcal{G}, \delta) \tag{5}$$

The superscript T denotes the transpose and $\boldsymbol{\lambda}$ is the Lagrange multipliers the vector of adjoint variables $\boldsymbol{\lambda} = [U^*{}_x, U^*{}_y, U^*{}_Z, p^*, T^*]$ computed to eliminate the influence of flow variables by solving the equation:

$$\frac{\partial \mathcal{F}^T}{\partial \delta} = -\boldsymbol{\lambda}^T \frac{\partial \mathcal{R}}{\partial \delta}\partial \delta \tag{6}$$

The solution of the above equation ensures iteratively that the objective function of an updated design will be solved by satisfying all the constraint conditions, i.e., the updated design of the VG will meet the flow conditions and the objective function defined.

Local Analysis of flow structure in the channel

The vortical structures of the RVG generated laterally by f_1 between P8 and P15 have lower velocity values than the vortical structures produced by the DWP in the same position. The above can be observed in figure 1 and represents a contribution to reducing the pressure drop in the channel. The local fluid flow temperature fields for all the channel RVG designs are illustrated in figure 2. Looking at the temperature fields at P15 for the optimal f_1 design, the colder fluid zones are present spanwise in the RVG cf. DWP RVG.

Figure 3a shows the spanwise Nusselt number averaged in the z-direction for the DWP, TPF morph and the two extreme designs obtained by setting $a = 1, b = 0$ and $a = 0, b = 1$ in the f_2 objective function. For the $a = 1, b = 0$ case, it is clear that it has the highest Nu_x values spanning from $x/h = -2$ to $x/h = 2$. Compared to all the other designs, this design increases the flow blockage area by increasing the frontal area near the leading edge of the VG. As a result, this will intensify the strength of main counter-rotating vortices, and the case will be characterised by higher peaks in the Nu_x distribution. Moreover, another interesting observation is the effect of increasing the frontal area near the trailing edge of the VG on spreading the corner vortices to a wider region. In the Nu_x distribution the $a = 1, b = 0$ case has a region of influence that spans from $x/h = -2$ to $x/h = 2$ compared to the DWP case, which spans from $x/h = -1.75$ to $x/h = 1.75$. For the TPF Morph case, the magnitude of the Nusselt spanwise distribution is smaller than the original DWP case near the channel centre and at the primary vortex location due to the decrease in the frontal area near the leading edge of the VG. However, this design shows the highest region of influence, covering approximately all the channel widths. Like the $a = 1, b = 0$ case, the TPF Morph increases its frontal area near the trailing edge; however, the VGs added concave curvature in the streamwise direction. The curved surface of the VG will guide the fluid to reach further lateral distance, and, in turn, two peaks are located in $x/h = \pm 1.75$ and $x/h = \pm 2.5$. The effect of decreasing the frontal area near the leading edge and streamlining the VG by creating a smooth curvature in front of the fluid reduces the friction factor below the friction factors of DWP and the $a = 1, b = 0$ cases, as shown in figure 3(c). The case $a = 0, b = 1$ shows the lowest Nu_x distribution due to the decrease in the frontal area near both the leading and trailing edges, resulting in the smallest friction factor distribution as shown in figure 3c. Finally, the Nu_x distribution is more significant than an empty channel in all configurations.

Figure 3b shows the streamwise x-averaged Nusselt number for all the cases studied. For all RGV cases, a peak is observed near the trailing edge of the VGs at $z/h = 2.75$. The highest value corresponds to the $a = 1, b = 0$ case, which yields a 95% enhancement in heat transfer of an empty channel. This highest enhancement is followed by two peaks related to the TPF morph and the DWP cases with approximately similar magnitudes resulting in an enhancement of 54% concerning an empty channel. Nevertheless, the TPF morph compensates for this similarity in Nu_z magnitudes at the trailing edge with an apparent higher enhancement at $z/h = 9$ than the DWP case, which explains the higher global Nusselt number for the TPF case. The Nu_z increase at $z/h = 9$ corresponds to a broader region occupied by the vortices and stronger induced vortices near the channel centre than in the DWP case. At $z/h = 9$, the TPF Morph case exhibits a 38% enhancement in heat transfer, while the DWP has a 17% enhancement compared to an empty channel. The $a = 0, b = 1$ case displays the lowest enhancement in heat transfer along all the channel lengths with a 21% enhancement at the trailing edge of RVG.

The streamwise friction factor distribution f_z is also presented in figure 3d for all the cases. At the channel's outlet, the friction factor displays an increase of pressure drop by 177% when compared to an empty channel for the $a = 1, b = 0$ case, a 97% increase for the DWP case, a 72% increase for the TPF case and finally, the lowest 21% increase of pressure drop for the $a = 1, b = 0$ case. In conclusion, the TPF morph case benefits from two main geometric modifications, a decrease in the front area and VG streamlining, by creating a frontal concave surface that finally results in moderate pressure drops while maintaining an enhanced heat transfer performance. For the $a = 1, b = 0$, the frontal area increase and sharp edges and corners in the RVG configuration greatly enhance heat transfer, however, at the expense of a larger pressure drop.

Figure 4 shows the spanwise Nusselt number distribution at several z/h positions. The spanwise vortical swirling strength S_{xz} is also presented in figure 5 to link the heat transfer enhancement mechanism with the flow structures. The swirling strength criterion is a popular vortex extraction approach developed by Zhou et al.[4] that can detect vortical structure where the maximum local value is associated with the vortical centre. In figure 4a, we can observe several peaks of Nu_{xz} in the spanwise direction. The first two peaks located at $x/h = \pm 1.25$ for the DWP design, at $x/h = \pm 1.475$ for the TPF morph design, at $x/h = \pm 1.425$ for the $a = 1, b = 0$ case and at $x/h = \pm 0.85$ for the $a = 0, b = 1$ case, show the effect of the counter-rotating pair of longitudinal

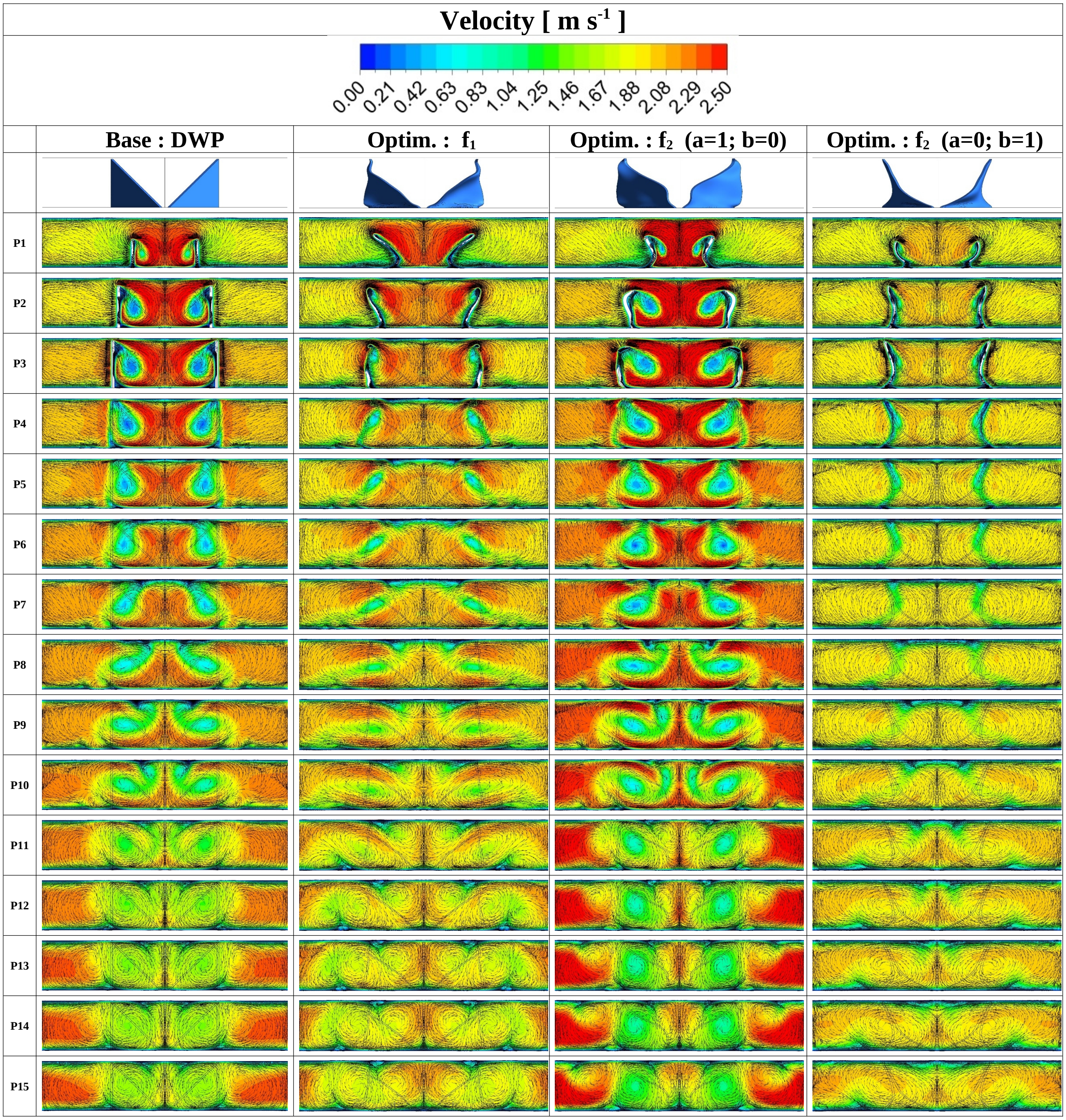

Figure 1. Streamwise velocity fields for base DWP design, optimum design using f_1, optimum design using f_2 with $a = 1$ & $b = 0$ and optimum design using f_2 with $a = 0$ & $b = 1$.

vortices on the heat transfer enhancement. Another two important peaks are located near the center of the channel, mainly at $x/h = \pm 0.5$ for the DWP case, at $x/h = \pm 1$ for the TPF morph case, at $x/h = \pm 0.725$ for the $a = 1, b = 0$ case and with the absence of such peaks in the $a = 0, b = 1$ case. These two peaks are influenced by the induced vortices created by the interaction between the main longitudinal vortices and the flowing core fluid passing near the centre-line of the channel. The $a = 1, b = 0$ case exhibits the highest peak since it is deformed in such a way as to increase the blockage area by decreasing the separating distance between the pair of VGs near their leading edge.

Other peaks are located at $x/h = \pm 1.6$ for the DWP case, $x/h = \pm 1.85$ for both the TPF morph and $a = 1, b = 0$ cases and

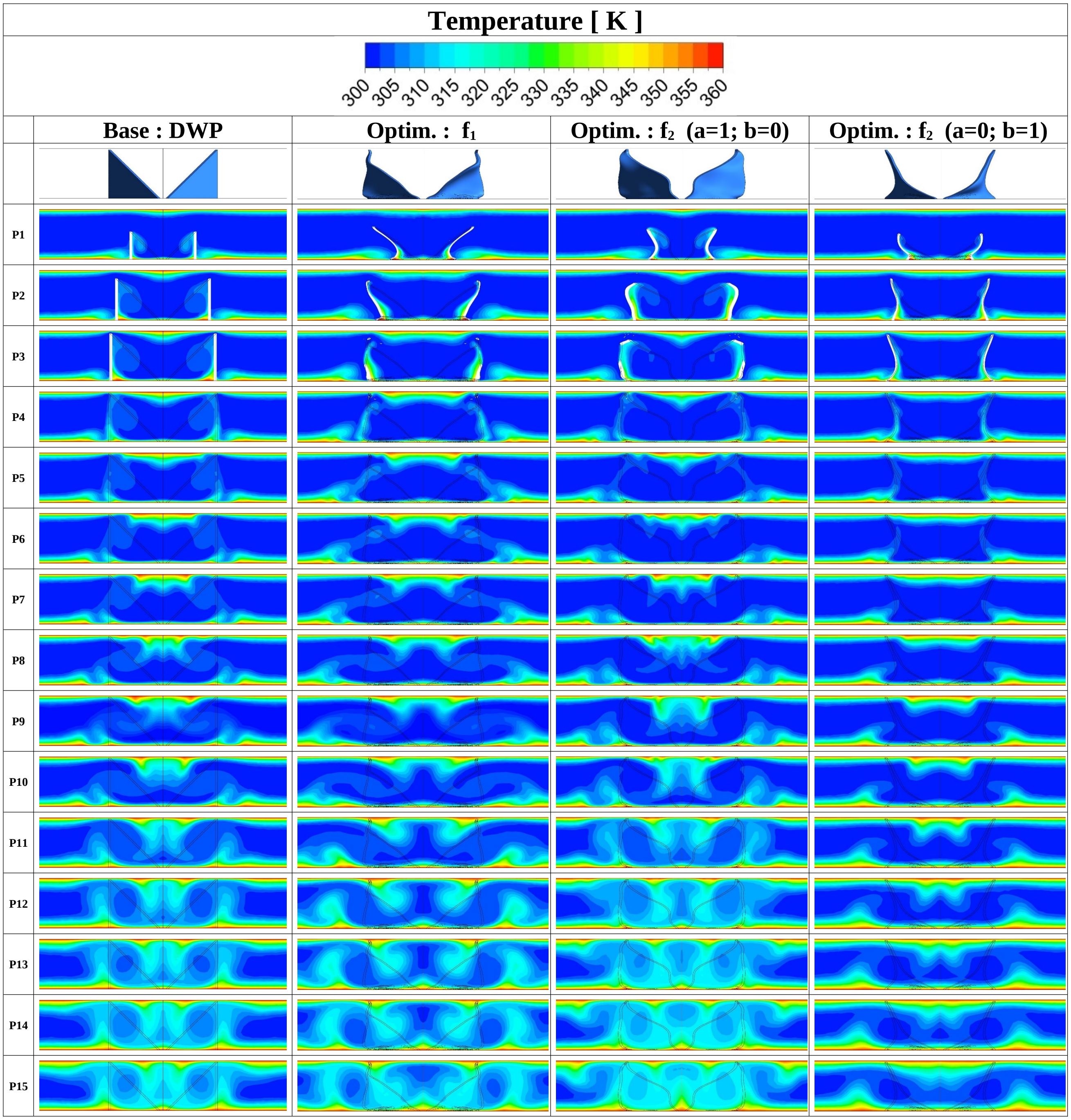

Figure 2. Streamwise temperature fields for base DWP design, optimum design using f_1, optimum design using f_2 with $a = 1$ & $b = 0$ and f_2 with $a = 0$ & $b = 1$.

finally at $x/h = \pm 1.1$ for the $a = 0, b = 1$ case. These peaks are associated mainly with the corner vortices created due to the development of an adverse pressure gradient when the fluid reaches the trailing edge of the RVG and moves in the shape of a horseshoe vortex.

We tracked the vortical swirling strength from the streamwise position $z/h = 3.1$ (figure 5a) to $z/h = 11.5$ (figure 5d) and found the evolution of the three types of vortices: A pair of induced vortices; a pair of main longitudinal vortices; and a pair of corner vortices, highlighted by six peaks in the swirling strength values. The $a = 1, b = 0$ case displays the highest values of swirling strength for all the types of vortices. However, for the TPF morph case, the main vortices show approximately the same swirling magnitudes as the DWP case but with a clear advantage regarding the higher magnitude of swirling strength

related to the induced vortical structures. These induced vortical structures in the TPF case cover a distance from $x/h = 0$ to $x/h = \pm 0.75$, as shown in figures 5b, c and d.

Furthermore, the $a = 1, b = 0$ case displays the highest Nu_{xz} distribution, as shown in figures 4b, c and d near the centre of the channel, mainly from $x/h = 0$ to $x/h = \pm 0.75$, which is caused by the highest swirling strength of the induced vortices. Other apparent peaks of Nu_{xz} for the $a = 1, b = 0$ case are located from $x/h = \pm 1$ to $x/h = \pm 2$, which are influenced by the main and corner vortices. On the other hand, the TPF morph case displays higher Nu_{xz} in the corners due to the effect of the widely spread corner vortices. For example, this is shown in figure 4d for the TPF case. The peaks in Nu_{xz} stretch from $z/h = \pm 1.5$ to approximately $z/h = \pm 2.5$, which is wider than all the other cases. In all the cases, a decrease in Nu_{xz} magnitude is noticed in the streamwise direction, which is related to the decrease in swirling strength caused by the dense dissipation effect.

Figure 6a shows the spanwise z-averaged swirling strength for all the cases studied. In dissecting the swirling strength for each type of vortical structure, the $a = 1, b = 0$ case has a counter-rotating pair at an average position of $x/h = \pm 0.9$ exhibits an increase of swirling strength of about 24% concerning the DWP case. The induced vortices located at $x/h = 0.25$ for the $a = 1, b = 0$ case show a rise of 200% of swirling strength compared to the induced vortices of the DWP case. Similarly, the corner vortices at an average position of $x/h = \pm 1.6$ show an increase of 67% of swirling strength for the $a = 1, b = 0$ case compared to the DWP corner vortices swirling power.

For the TPF morph case, the swirling strength of the primary vortices located at the average position of $x/h = \pm 1.3$ displays a reduction in swirling power of about 22% compared to the primary vortices of the DWP case. An enhancement of 115% compared to the DWP-induced vortices occurs through the swirling strength of the induced vortices, located at $x/h = \pm 0.525$. Finally, the corner vortices show approximately the same order of magnitude for the DWP case with a shift of propagation towards the corners of the channel. For the $a = 0, b = 1$ case, two peaks correspond to the pair of primary vortices with an apparent absence of induced and corner vortices in this case. The primary vortices located at $x/h = \pm 0.97$ show a reduction of 66% of swirling strength concerning the primary vortices of the DWP case, which explains the relatively low heat transfer enhancement and low-pressure drop for this case.

Finally, we compare the streamwise x-average swirling strength for all the cases in figure 6b. A peak is observed downstream of the RVG in all the cases with the highest swirling strength corresponding to the $a = 1, b = 0$ case, followed by the DWP case, the TPF morph case and the $a = 0, b = 1$ case, which has the lowest swirling strength among all. An important observation is that the streamwise decrease in swirling power is more substantial in the $a = 1, b = 0$ compared to the other cases. This is due to the relatively more considerable reduction of the primary vortices strength seen, for example, in the spanwise evolution of swirling power in figures 5. This steepest decrease is the closeness between the primary vortices, the induced vortices and the channel walls, which accelerates their dissipation. From a global point of view, integrating the streamwise swirling strength over all the channel length and comparing it with the DWP case, the $a = 1, b = 0$ shows an increase of 53% of swirling power, the TPF case shows an increase of 6% and the $a = 0, b = 1$ shows a reduction of 61% of swirling strength.

References

1. Schramm, Matthias and Stoevesandt, Bernhard and Peinke, Joachim. Optimization of Airfoils Using the Adjoint Approach and the Influence of Adjoint Turbulent Viscosity. *Computation* **6**, DOI: 10.3390/computation6010005 (2018).

2. Mengze Wang and Qi Wang and Tamer A. Zaki. Discrete adjoint of fractional-step incompressible Navier-Stokes solver in curvilinear coordinates and application to data assimilation. *J. Comput. Phys.* **396**, 427–450, DOI: 10.1016/j.jcp.2019.06.065 (2019).

3. Roth, R. and Ulbrich, S. A Discrete Adjoint Approach for the Optimization of Unsteady Turbulent Flows. *Flow Turbul. Combust* **90**, 763–783, DOI: 10.1007/s10494-012-9439-3 (2013).

4. ZHOU, J., ADRIAN, R. J., BALACHANDAR, S. & KENDALL, T. M. Mechanisms for generating coherent packets of hairpin vortices in channel flow. *J. Fluid Mech.* **387**, 353–396, DOI: 10.1017/S002211209900467X (1999).

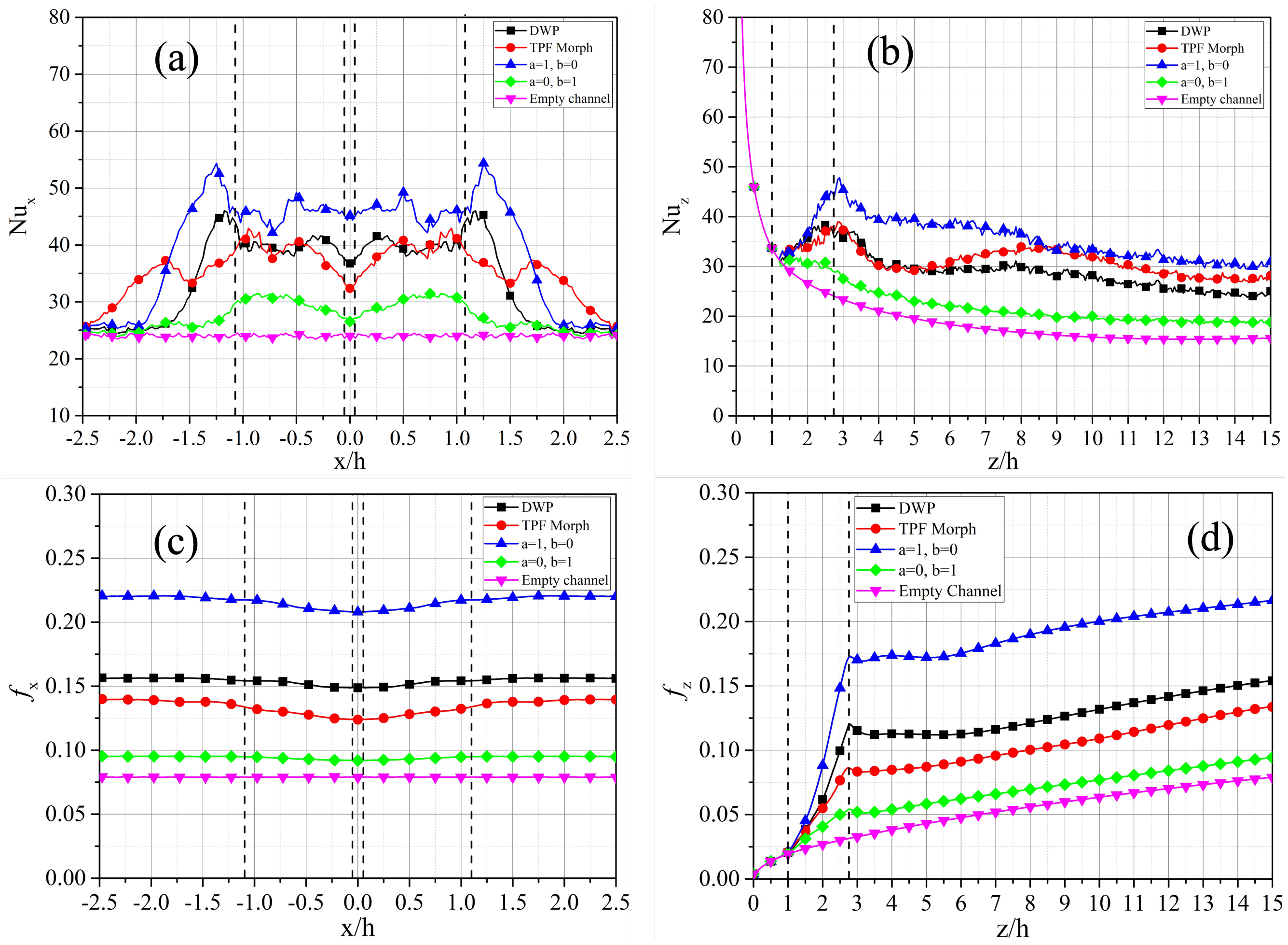

Figure 3. Optimization results of: (a) Spanwise z-averaged Nusselt number Nu_x; (b) Streamwise x-averaged Nusselt number Nu_z; (c) Spanwise z-averaged friction factor f_x; (d) Streamwise x-averaged friction factor f_z. Comparisons between an empty channel, the DWP base design and the three adjoint-based optimum designs obtained with a TPF Morph objective f_1, extreme objective f_2 $(a = 1; b = 0)$ and extreme objective f_2 $(a = 0; b = 1)$.

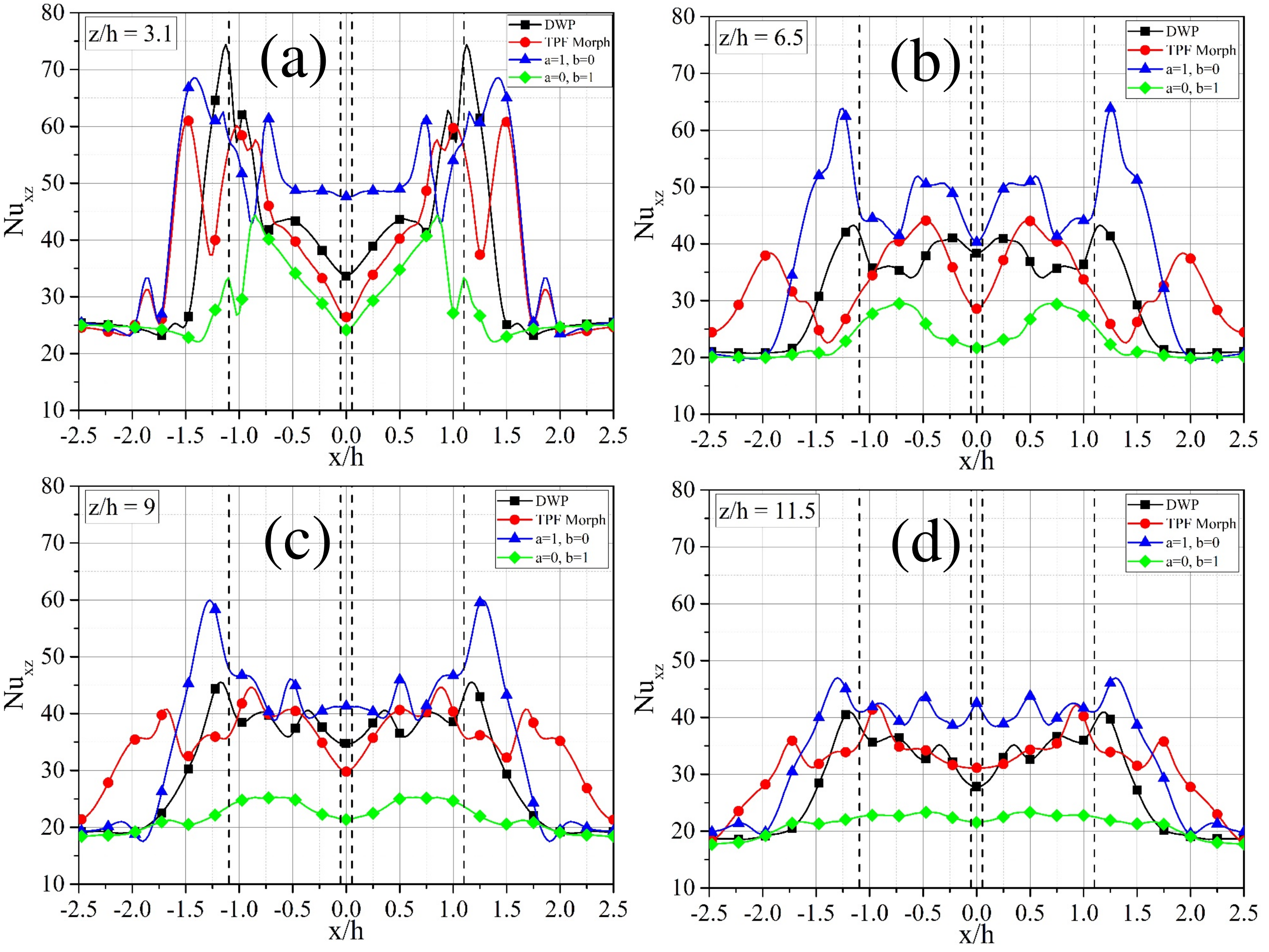

Figure 4. Optimization results of: (a) Spanwise Nu_{xz} at $z/h = 3.1$; (b) Spanwise Nu_{xz} at $z/h = 6.5$; (c) Spanwise Nu_{xz} at $z/h = 9$; (d) Spanwise Nu_{xz} at $z/h = 11.5$. Comparisons between the DWP base design and the three adjoint-based optimum designs obtained with a TPF Morph objective f_1, extreme objective f_2 $(a = 1; b = 0)$ and extreme objective f_2 $(a = 0; b = 1)$.

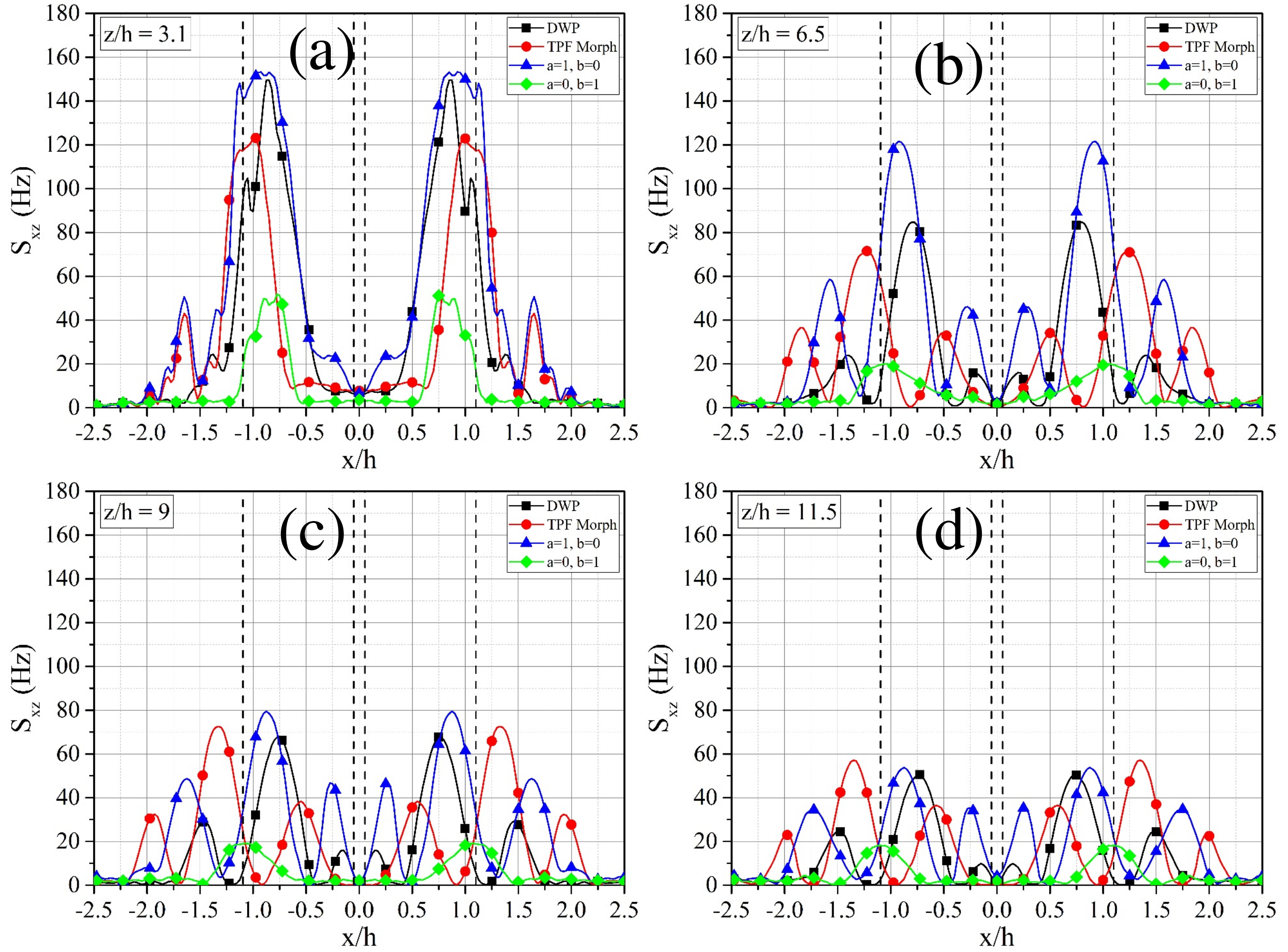

Figure 5. Optimization results of: (a) Spanwise S_{xz} at $z/h = 3.1$; (b) Spanwise S_{xz} at $z/h = 6.5$; (c) Spanwise S_{xz} at $z/h = 9$; (d) Spanwise S_{xz} at $z/h = 11.5$. Comparisons between the DWP base design and the three adjoint-based optimum designs obtained with a TPF Morph objective f_1, extreme objective f_2 $(a = 1; b = 0)$ and extreme objective f_2 $(a = 0; b = 1)$.

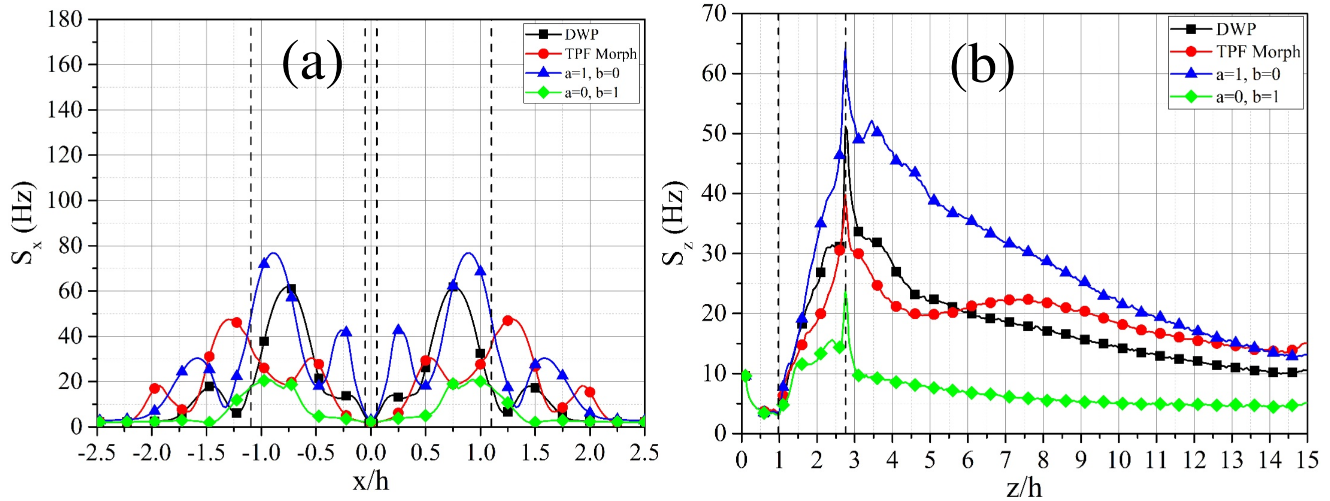

Figure 6. Optimization results of: (a) Spanwise S_x; (b) Streamwise S_z. Comparisons between the DWP base design and the three adjoint-based optimum designs obtained with a TPF Morph objective f_1, extreme objective f_2 $(a = 1; b = 0)$ and extreme objective f_2 $(a = 0; b = 1)$.
